# *In Vitro* Interaction of *Pseudomonas aeruginosa* with Human Middle Ear Epithelial Cells

**DOI:** 10.1371/journal.pone.0091885

**Published:** 2014-03-14

**Authors:** Rahul Mittal, M’hamed Grati, Robert Gerring, Patricia Blackwelder, Denise Yan, Jian-Dong Li, Xue Zhong Liu

**Affiliations:** 1 Department of Otolaryngology, University of Miami Miller School of Medicine, Miami, Florida, United States of America; 2 Center for Advanced Microscopy, University of Miami, Coral Gables, Florida, United States of America; 3 RSMAS, University of Miami, Key Biscayne, Florida, United States of America; 4 Center for Inflammation, Immunity, and Infection and Department of Biology, Georgia State University, Atlanta, Georgia, United States of America; 5 Department of Human Genetics, University of Miami Miller School of Medicine, Miami, Florida, United States of America; University of North Dakota, United States of America

## Abstract

**Background:**

Otitis media (OM) is an inflammation of the middle ear which can be acute or chronic. Acute OM is caused by *Streptococcus pneumoniae, Haemophilus influenzae, and Moraxella catarrhalis* whereas *Pseudomonas aeruginosa* is a leading cause of chronic suppurative otitis media (CSOM). CSOM is a chronic inflammatory disorder of the middle ear characterized by infection and discharge. The survivors often suffer from hearing loss and neurological sequelae. However, no information is available regarding the interaction of *P. aeruginosa* with human middle ear epithelial cells (HMEECs).

**Methodology and Findings:**

In the present investigation, we demonstrate that *P. aeruginosa* is able to enter and survive inside HMEECs via an uptake mechanism that is dependent on microtubule and actin microfilaments. The actin microfilament disrupting agent as well as microtubule inhibitors exhibited significant decrease in invasion of HMEECs by *P. aeruginosa*. Confocal microscopy demonstrated F-actin condensation associated with bacterial entry. This recruitment of F-actin was transient and returned to normal distribution after bacterial internalization. Scanning electron microscopy demonstrated the presence of bacteria on the surface of HMEECs, and transmission electron microscopy confirmed the internalization of *P. aeruginosa* located in the plasma membrane-bound vacuoles. We observed a significant decrease in cell invasion of *OprF* mutant compared to the wild-type strain. *P. aeruginosa* induced cytotoxicity, as demonstrated by the determination of lactate dehydrogenase levels in culture supernatants of infected HMEECs and by a fluorescent dye-based assay. Interestingly, *OprF* mutant showed little cell damage compared to wild-type *P. aeruginosa*.

**Conclusions and Significance:**

This study deciphered the key events in the interaction of *P. aeruginosa* with HMEECs *in vitro* and highlighted the role of bacterial outer membrane protein, OprF, in this process. Understanding the molecular mechanisms in the pathogenesis of CSOM will help in identifying novel targets to design effective therapeutic strategies and to prevent hearing loss.

## Introduction

Chronic suppurative otitis media (CSOM) is a frequently encountered chronic inflammation of the middle ear and mastoid process characterized by both tympanic membrane perforation and discharge [1]. CSOM is one of the most common chronic infectious diseases worldwide. CSOM affects diverse racial and cultural groups in both developing and developed countries and occurs frequently in children [2]. When it occurs during the first two years of life, the consequent hearing loss is likely to have serious effects on the critical period of a young child’s development, and may have long term effects on language development, early communication, auditory processing, psychosocial and cognitive development, as well as educational progress and achievement [3], [4]. CSOM has been associated with considerable morbidity and substantial healthcare costs [5]. Without treatment, there is continuous or intermittent purulent ear discharge for months or even years with destruction of the bones of the middle ear and increasing hearing impairment [6]. The presence of mucus prevents the transmission of sound waves from middle ear to inner ear leading to conductive hearing loss. Chronic infection of the middle ear leads to oedema of the middle-ear lining and discharge, tympanic membrane perforation, and possibly ossicular chain disruption that further aggravates the problem of hearing loss in CSOM patients [7]. CSOM can also cause sensorineural hearing loss [8]–[10]. It has been shown that inflammatory mediators generated during CSOM can penetrate from the round window into the inner ear causing loss of hair cells in the cochlea leading to sensorineural hearing loss in animal models [11]–[13]. Human studies have also demonstrated the loss of outer and inner hair cells in the basal turn of the cochlea in CSOM patients [14]. The pathogenesis of CSOM is multifactorial including abnormal function of the eustachian tube (resulting from small size, genetic syndromes, viral respiratory infections, functional immaturity, allergy, and environmental smoke exposure), invasion of the middle ear by bacteria and/or viruses, and inflammation [15], [16]. The bacterial infection of the middle ear is the most common cause of CSOM. Antibiotics and surgery are the only treatment options for CSOM, but have only moderate efficacy against the disease. The excessive use of the antibiotics has led to the emergence of resistant bacteria that has further complicated the treatment of CSOM. Antibiotics can also have severe ototoxic effects, especially in children, which should also be taken into consideration [17], [18]. In addition, antibiotics cause lysis of bacteria with subsequent release of endotoxin and consequent triggering of inflammatory processes that can further aggravate inflammation. Therefore, alternative treatment strategies against CSOM are warranted for which understanding the pathogenesis of disease is of utmost importance.

The colonization of host mucosal surfaces is the first and essential step in the infectious process [19]. The infection of a host by a pathogenic microorganism triggers complex cascades of events that influence the immediate and long-term outcome of this interaction [20]–[22]. One of the most important initial signaling events involves interaction of epithelial cells with the pathogen [23], [24]. The surface exposed moieties on pathogens like outer membrane proteins (OMPs) have been shown to play an important role in mediating this interaction [25], [26]. The middle ear is lined by a layer of epithelial cells which acts as a physical barrier and forms an important line of host defense [27]. Human middle ear epithelial cells (HMEECs) have been demonstrated to secrete diverse molecules in response to stimulation like whole bacteria, bacterial products or lipopolysaccharide (LPS), providing efficient protection against infectious diseases [28]–[31]. This interplay between HMEECs and bacteria can have a profound influence on the ultimate outcome of infection during CSOM.

*Pseudomonas aeruginosa* is the most frequently isolated pathogen in CSOM reported from different parts of the world [32]–[37]. *P. aeruginosa* induced CSOM is characterized by the presence of numerous bacteria, inflammatory cells, middle ear effusion, and middle ear epithelial cell injury. *P. aeruginosa* possess a plethora of virulence factors which facilitate the ability of this pathogen to cause a diverse array of infections in humans [38]. However the lack of OprF expression severely hampers the ability of *P. aeruginos*a to cause infections. OprF is a general porin of *P. aeruginosa* which facilitates the nonspecific diffusion of ionic particles and small polar nutrients [39]. OprF belongs to a class of proteins which are proposed to have a wide range of functions. This class includes diverse proteins such as MotB, a cytoplasmic membrane protein which is part of the flagellar rotation assembly in *Escherichia coli* and *Bacillus subtilis*. OprF has also been demonstrated to play an important role in adhesion of *P. aeruginosa* to human alveolar epithelial cells, glial cells and Caco-2/TC7 cells [40], [41]. *OprF* mutant caused only limited necrosis when inoculated in the middle veins of *Cichorium intybus* leaves, even after 8 days compared to wild-type strain which caused significant necrosis [41]. OprF has also been implicated in the ability of *P. aeruginosa* to form biofilms under anaerobic conditions [42]. However the role of OprF in CSOM is not known.

Although *P. aeruginosa* accounts for the majority of cases of CSOM, no information is available regarding the interaction of this pathogen with HMEECs. In the present study, we examined the interaction of *P. aeruginosa* with HMEECs. We observed that *P. aeruginosa* invades HMEECs and ultimately causes cell damage for which OprF expression is required.

## Materials and Methods

### Bacterial strains

The bacterial strains used in this study were *P. aeruginosa* H103 (PAO1 wild-type prototroph), an *oprF* mutant of H103 strain (H636), and pOprF (H636O) which corresponds to H636 strain complemented by plasmid pRW5 consisting of the functional *oprF* gene from *P. aeruginosa* H103 cloned into pUCP19 [41], [43]. Bacteria were grown at 37°C in a rotary shaker in the presence of appropriate antibiotics, as described earlier [41], [43]. *oprF* mutant and pOprF (H636O) were grown in the presence of streptomycin and carbenicillin, respectively.

### Cell Culture

Human middle ear epithelial cells (HMEECs) (kindly provided by Dr. David Lim) were generated from human middle ear mucosa as described earlier [27]. HMEECs used in our studies were no more than six passages. HMEECs were cultured and maintained as described earlier [27]–[31]. Briefly, HMEECs were cultured in a 1∶1 mixture of Bronchial Epithelial Cell Basal Medium (Lonza, Allendale, NJ) and Dulbecco’s Modified Eagle Medium (Cellgro, Manassas, VA) supplemented with bronchial epithelial growth medium (BEGM) Singlequots (Lonza, Allendale, NJ) and 10% fetal bovine serum (Life Technologies, Carlsbad, CA).

### Invasion assays

Gentamicin protection assays were used to quantify the extent of bacterial invasion of HMEECs. Briefly, HMEECs were infected with bacteria at various multiplicity of infection (MOI) and for different time-periods. After incubation, the cells were washed 5 times with warm RPMI followed by addition of medium containing gentamicin (200 μg/ml) and further incubated for 1 h at 37°C. The cells were washed 3 times with RPMI and then lysed with 1% saponin to release intracellular bacteria. Serial dilutions were then plated on blood agar plates and bacterial colonies were counted the next day. The binding of bacteria to HMEECs was determined by lysing the cells without adding gentamicin. In some experiments, bacteria were pretreated with monoclonal anti-OprF antibody (kindly provided by Dr. Hancock) and then used in the invasion assay. The monoclonal antibody (mAb) was specific to surface epitopes of OprF and was generated as described previously [44], [45]. HMEECs were also pretreated with different concentrations of purified exogenous OprF. OprF was purified from *P. aeruginosa* as described earlier and purity was confirmed by western blotting [40]. To determine the effect of cytoskeletal inhibitors, HMEECs were pretreated with different concentrations of cytochalasin D, vinblastine, nocodazole or colchicine for 30 min before infecting with bacteria and maintained in the medium for the entire infection period.

### Scanning electron Microscopy (SEM)

HMEECs were cultured on glass cover slips and were infected with bacteria for varying time periods. After incubation, the cells were washed 5 times with warm phosphate buffer saline (PBS) buffer to remove unbound bacteria and were then processed for SEM. Samples were fixed in 2% glutaraldehyde in PBS buffer followed by three changes of PBS buffer for 10 min each. The samples were then post–fixed in 1% osmium tetroxide in PBS buffer for 45 min and rinsed in three changes of PBS buffer for 10 min each. The samples were dehydrated in a graded series of ethanol, dried in hexamethyldisilazane (HMDS) and mounted on carbon adhesive tabs fixed to metal stubs. The samples were coated with palladium in a plasma sputter coater and viewed in a scanning electron microscope (FEI, ESEM-FEG XL-30).

### Transmission electron Microscopy (TEM)

HMEECs were infected with bacteria for varying time periods. After incubation, the cells were washed with PBS and fixed using 2% glutaraldehyde. The samples were rinsed in three washes of PBS buffer then post-fixed in 1% osmium tetroxide in 0.1 M phosphate buffer for 1 hour. After buffer rinses, specimens were dehydrated through a series of graded ethanol, placed in two rinses of propylene oxide for 5 minutes each and then put in a 1:1 mixture of propylene oxide: EMbed/Araldite resin (Electron Microscopy Sciences, Fort Washington, PA) for overnight at room temperature. Next day, the pellets were placed in fresh EMbed/Araldite and put in a vacuum desiccator for 2–4 hours. The samples were changed to fresh EMbed/Araldite and polymerized overnight. Silver/gold sections were then cut on a Leica Ultracut E (Leica, Buffalo Grove, IL), stained in uranyl acetate and lead citrate, and viewed in a JEOL 1400 electron microscope (JEOL, Peabody, MA) with Gatan Orius SC1000 camera (Gatan, Pleasanton, CA).

### Immunofluorescence

For staining of bacteria and actin, HMEECs were cultured in 8-well chamber slides and infected with *P. aeruginosa* for varying time periods. After incubation, cells were washed three times with PBS buffer and then fixed and permeabilized with BD cytofix and cytoperm reagent (BD Biosciences, San Jose, CA) for 30 min. After washing, the cells were blocked with 3% normal goat serum (NGS) for 20 min and then incubated with anti-*Pseudomonas aeruginosa* antibody (Abcam, Cambridge, MA) for 45 min followed by Alexa Fluor 488 antibody (Life Technologies, Carlsbad, CA). After washing, cells were counterstained for actin with rhodamine phalloidin (Life Technologies, Carlsbad, CA) for 45 min, washed and mounted in an antifade Vectashield solution containing 4, 6-diamidino-2-phenylindole (DAPI) (Vector Laboratories, Burlingame, CA). The cells were viewed with a Zeiss LSM 710 microscope (Carl Zeiss, Germany) and images were assembled using Adobe photoshop 7.0.

### Lactate dehydrogenase (LDH) determination

The extent of cell damage upon *P. aeruginosa* infection was assessed by measurement of LDH release by eukaryotic cells upon cytoplasmic membrane destabilization. LDH is a stable cytosolic enzyme and has been used as an indicator of cell damage [46]–[48]. HMEECs were infected with bacteria at various MOI and for different time periods. LDH was then determined in culture supernatants of HMEECs using LDH kit as per manufacturer’s instructions (Cayman Chemical, Ann Arbor, MI). Uninfected monolayers were included as negative control. Maximum LDH release induced by treatment of cells with 1% Triton X-100 was used as positive control. Results were expressed as percentage LDH release compared to the positive control.

### Live/Dead Assay

HMEECs grown in 8-well chamber slides were infected with bacteria at various MOI and for different time-periods. Uninfected cells served as negative control. After incubation, the cells were washed with PBS buffer and then stained with LIVE/DEAD viability kit (Life Technologies, Carlsbad, CA) according to the manufacturer’s instructions. The fluorescent staining was viewed under a Zeiss LSM-710 laser scanning microscope (Carl Zeiss, Germany) and images were assembled using Adobe photoshop 7.0.

### Statistical analysis

Statistical significance was determined by a paired, two-tailed Student’s t test using SPSS software. Values of p < 0.05 were considered to be statistically significant.

## Results

### *P. aeruginosa* invades HMEECs

**Figure 1:**
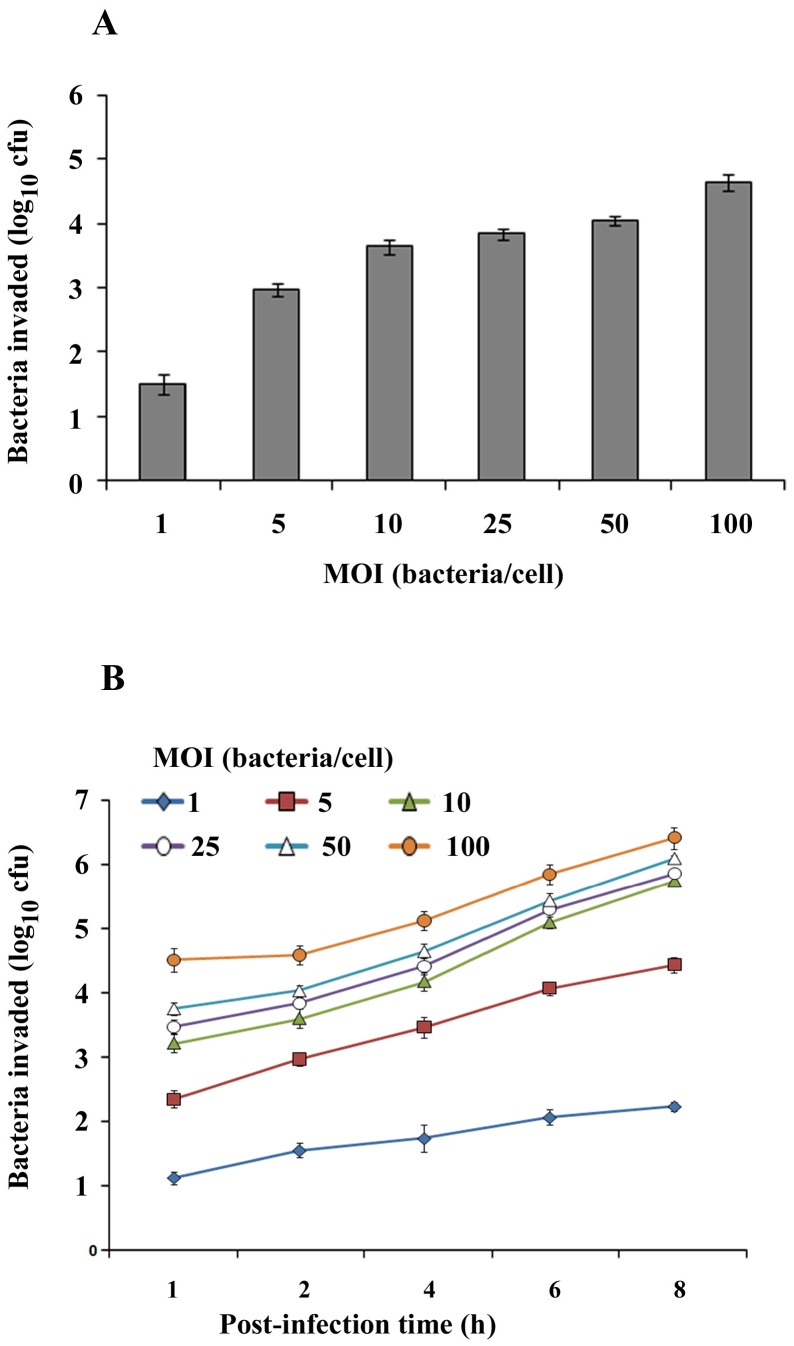
Invasion of *P. aeruginosa* in HMEECs is dose and time dependent. HMEECs were infected with *P. aeruginosa* at different MOI for 2h and invasion was determined by gentamicin protection assay (A). In separate experiments, HMEECs were infected with *P. aeruginosa* at an MOI of 10 for varying time periods and bacterial invasion was determined by gentamicin protection assay (B). Data represents mean ± SD. Results are representative of five independent experiments carried out in triplicate.

To determine whether *P. aeruginosa* invades HMEECs, the cells were infected with bacteria at different MOI and gentamicin protection assay was performed. First, we examined the effect of inoculum size on bacterial invasion of HMEECs. By 2h post-infection, log 1.51 colony forming unit (cfu) bacteria were recoverable from HMEECs at an MOI of 1 (Figure 1A). With increase in MOI from 1 to 5, log 2.98 cfu bacteria invaded HMEECs. At an MOI of 10, log 3.64 cfu bacteria were demonstrable inside HMEECs. Further increase in MOI to 25 and 50 caused marginal increase in number of bacteria inside HMEECs. At higher MOI of 100, log 4.32 cfu bacteria were recoverable from the cells. We then examined whether increasing incubation time had any effect on bacterial cell invasion. At an MOI of 1, the number of bacteria inside HMEECs increased from log 1.15 cfu at 1h post-infection to 2.27 cfu by 8h post-infection whereas it increased from 2.38 cfu at 1h post-infection to 4.49 cfu at 8h post-infection at an MOI of 5 (Figure 1B). With increase in MOI to 10, the number of bacteria recoverable from HMEECs increased from 3.26 cfu at 1h post-infection to 5.80 cfu at 8h post-infection. Further increase in MOI to 25 and 50 caused marginal increase in bacteria at 8h post-infection compared to an MOI of 10. At higher MOI of 100, the number of bacteria increased from 4.56 cfu at 1h post-infection to 6.48 cfu by 8h post-infection. These results suggest that *P. aeruginosa* is able to enter and survive inside HMEECs.

**Figure 2:**
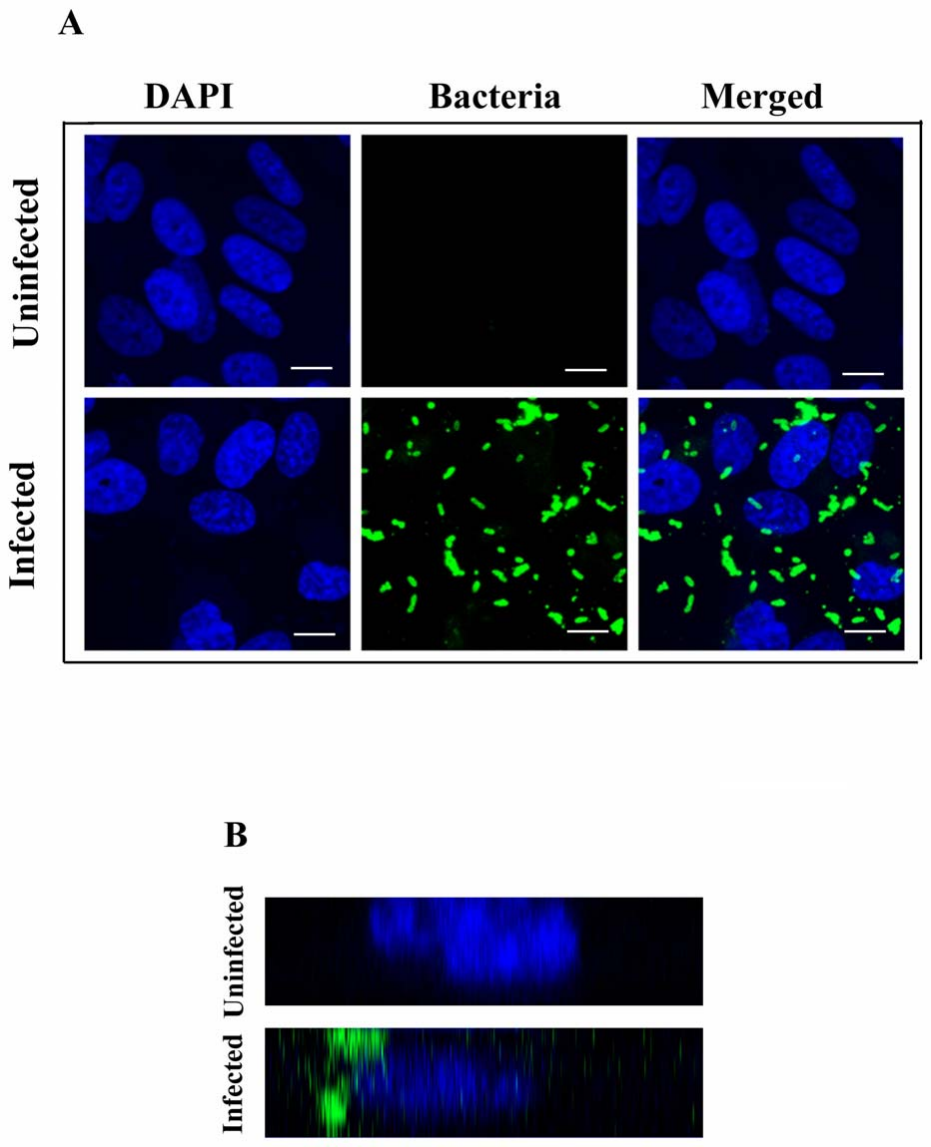
Confocal Microscopy of HMEECs infected with *P. aeruginosa*. HMEECs were infected with *P. aeruginosa* at an MOI of 10 for 2h and then bacteria were stained with anti-*P. aeruginosa* antibody followed by a secondary Alexa Fluor® 488 antibody. The slides were counterstained with 4’,6-diamidino-2-phenylindole (DAPI) and visualized by confocal laser fluorescence microscope (A). The analytical sectioning was performed from top to bottom of cells and orthogonal panels were prepared demonstrating bacterial invasion of HMEECs (B). Results are representative of four independent experiments carried out in triplicate. Scale bars 10 μm.

To further confirm the invasion data, we performed confocal microscopy of infected HMEECs. By 2h post-infection, the bacteria were observed closer to the nuclei of the cells indicating internalization (Figure 2A). Z-sectioning and 3-dimensional imaging of the cells infected with *P. aeruginosa* provided direct evidence of localization of bacteria inside cells (Figure 2B and Video S1).

### Electron Microscopy

**Figure 3:**
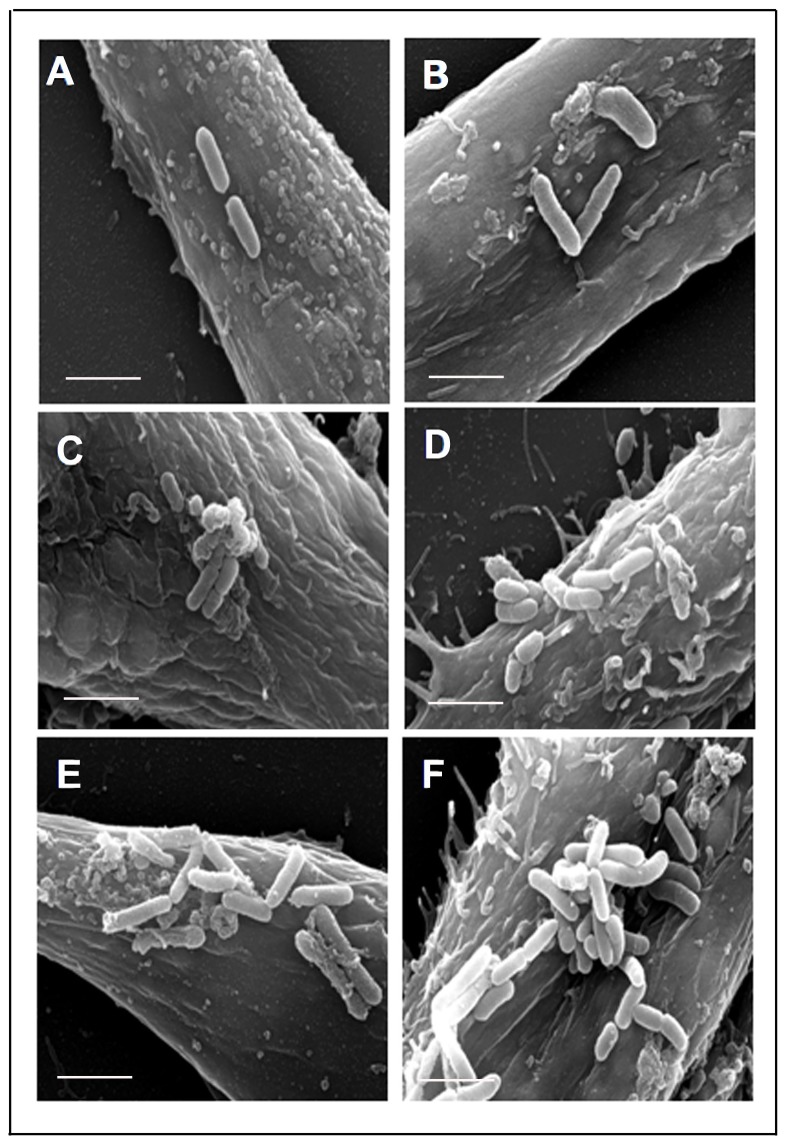
Scanning electron micrographs demonstrating interaction of *P. aeruginosa* with HMEECs. Epithelial cells were infected with *P. aeruginosa* for 30 min (A), 1h (B), 1.5h (C), 2h (D), 4h (E) and 8h (F) and then subjected to SEM. Large number of bacteria were seen on the surface of HMEECs at 8h post-infection. Results are representative of four independent experiments carried out in triplicate. Scale bars 2 μm.

**Figure 4:**
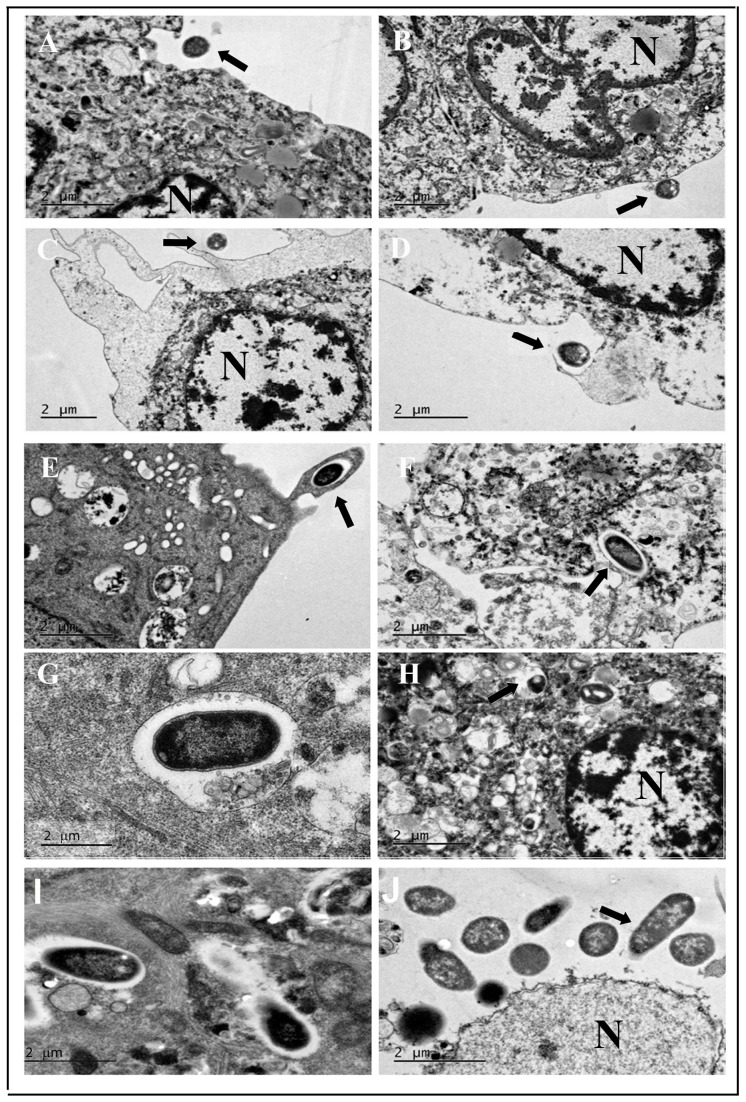
Transmission electron micrographs demonstrating internalization of *P. aeruginosa* in HMEECs. Epithelial cells were infected with *P. aeruginosa* for 15 min (A), 30 min (B-E), 1 h (F and G), 8h (H and I), 16h (J), and then subjected to TEM. Results are representative of four independent experiments carried out in triplicate. Arrows indicate bacteria. N: Nucleus. Scale bars 2 μm.

The ultrastructural interaction of *P. aeruginosa* with HMEECs was characterized by electron microscopy. HMEECs were infected with *P. aeruginosa* at an MOI of 10 for varying time periods and subjected to electron microscopy examinations. Scanning electron microscopy (SEM) exhibited the intimate association of *P. aeruginosa* with epithelial cells (Figure 3). There was increase in the numbers of adherent bacteria with increase in post-infection time period (Figure 3A-F). Few bacteria were visible on the surface of HMEECs at 30 min post-infection, but by 8h large clusters of adherent *P. aeruginosa* were demonstrable on the cell surface. Transmission electron microscopy revealed the sequential cell invasion of *P. aeruginosa.* At 15 min post-infection, bacteria were observed in close proximity to HMEECs followed by adhesion to cell surface at 30 min post-infection (Figure 4A and B). HMEECs extended appendages around the adherent bacteria with the formation of pseudopod-like structures (Figure 4C and D). Some of the bacteria were observable in HMEECs cell invaginations (Figure 4E). After internalization, bacteria were observed in the membrane bound vacuoles at 1h post-infection (Figure 4F). In the majority of the cells, one bacterium per vacuole was observed (Figure 4G). However at 8h post-infection, bacteria were observed to disrupt the membrane and exit the vacuoles (Figure 4H and I). A large number of free bacteria were demonstrable in the cytoplasm of cells at 16h post-infection (Figure 4J).

### Invasion of *P. aeruginosa* into HMEECs requires both microtubules and microfilaments

**Figure 5:**
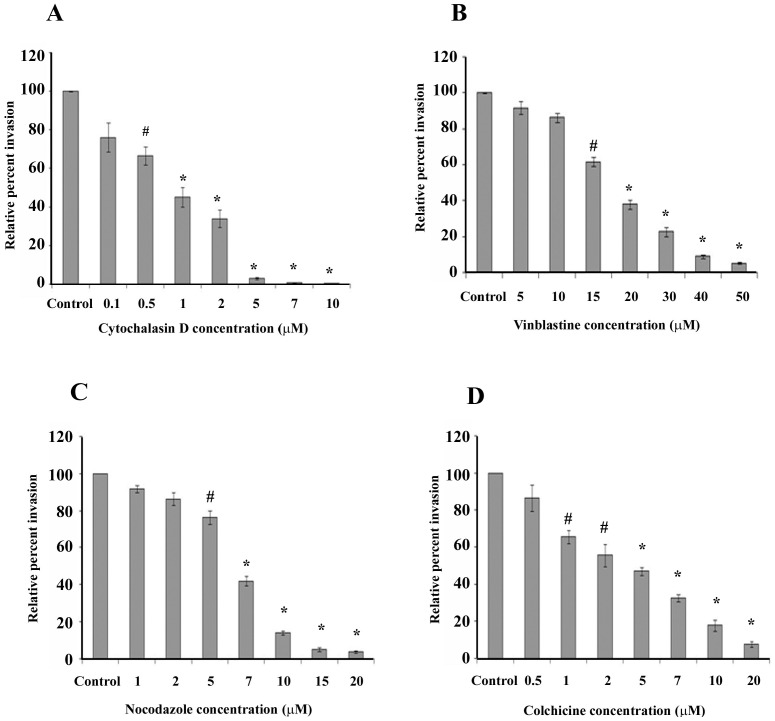
*P. aeruginosa* invasion of HMEECs is dependent on both microfilament and microtubule dependent uptake mechanisms. HMEECs were pretreated with different concentrations of cytochalasin D (A), vinblastine (B), nocodazole (C), or colchicine (D) for 30 min and then infected with *P. aeruginosa* at an MOI of 10 for 2h. Bacterial invasion was determined by gentamicin protection assay and results were expressed as percentage compared to the bacterial invasion in control cells. Data represents mean ± SD and is representative of five individual experiments carried out in triplicate. # P<0.05 or *P<0.001 compared to control.

To determine the role of microfilaments and microtubules in the invasion of HMEECs by *P. aeruginosa*, epithelial cells were pretreated with cytoskeleton dynamics inhibitors, cytochalasin D for microfilaments, and vinblastine, colchicine and nocodazole for microtubules. Cells were pretreated with various concentrations of cytochalasin D which causes microfilament depolymerization, and then infected with bacteria at an MOI of 10 for 2h. As these inhibitors were dissolved in dimethylsulfoxide (DMSO), therefore, HMEECs treated with DMSO alone served as control. With increase in concentration of cytochalasin D, there was a significant decrease in the bacterial invasion compared to control cells. HMEECs pretreated with 1 μM of cytochalasin D showed 55% decrease in bacterial invasion whereas cells pretreated with 10 μM showed >98% inhibition compared to control cells (P<0.001) (Figure 5A). Similar decrease in cell invasion was observed following pretreatment of HMEECs with vinblastine, colchicine and nocodazole, each of which cause microtubule disruption. Pretreatment of HMEECs with 1 μM of colchicine resulted in 34% decrease in bacterial invasion, whereas there was 92% decrease in invasion as compared to control when cells were pretreated with 20 μM of colchicine (P<0.001) (Figure 5B). Nocodazole pretreated cells demonstrated >85% decrease in *P. aeruginosa* invasion as compared to control at concentrations between 10–20 μM (P<0.001) (Figure 5C). Vinblastine was effective in inhibiting cell invasion of *P. aeruginosa* by 90–95% at concentrations of 40 to 50 μM (Figure 5D). We observed that there were no toxic effects of these reagents on bacteria or on cells at the tested concentrations (data not shown). These results suggests that *P. aeruginosa* invades HMEECs through both microfilament and microtubule dependent uptake mechanisms.

### *P. aeruginosa* induces actin cytoskeleton changes in HMEECs

**Figure 6:**
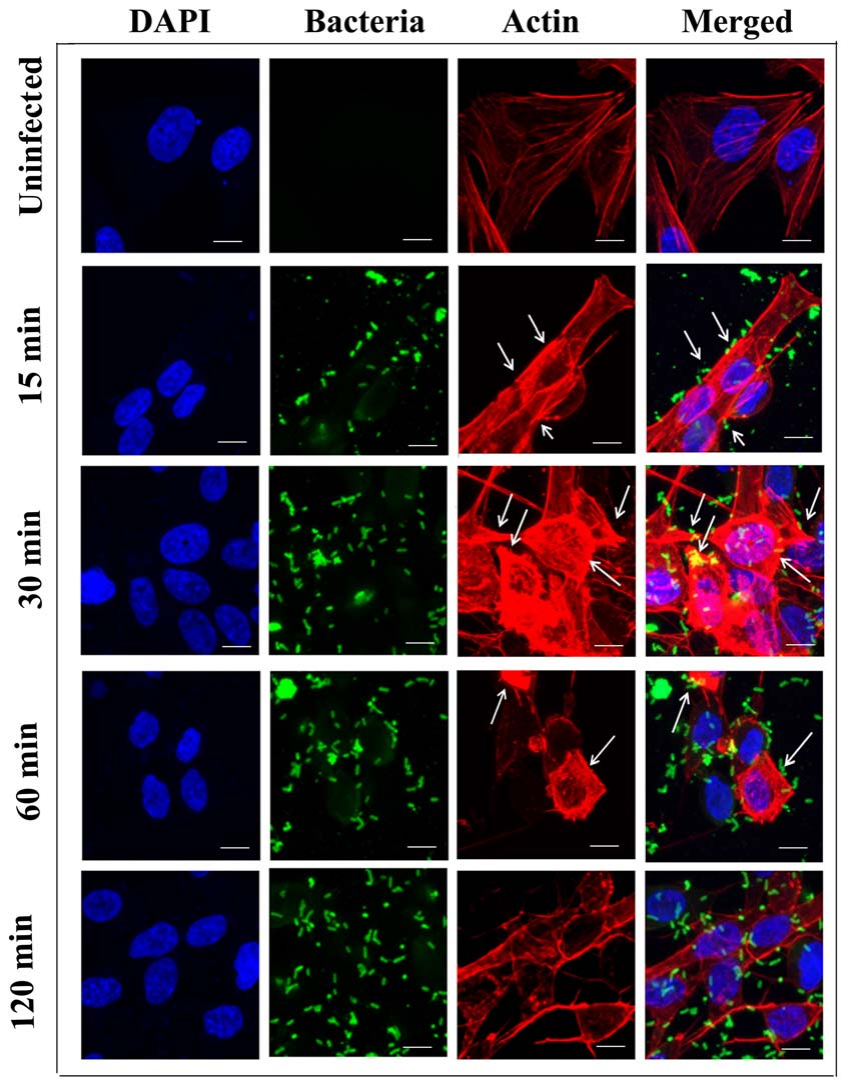
*P. aeruginosa* induces cytoskeletal rearrangements during invasion of HMEECs. Cells were either left uninfected or infected with *P. aeruginosa* at an MOI of 10 for 15 min, 30 min, 60 min and 120 min. Bacteria were stained with anti-*P. aeruginosa* antibody followed by a secondary Alexa Fluor® 488 antibody. Actin was stained with rhodamine phalloidin and then mounted in medium containing DAPI. Results are representative of four independent experiments carried out in triplicate. Arrows indicate actin accumulation. Scale bars 10 μm.

Pathogens have been demonstrated to manipulate host cell cytoskeleton to facilitate their entry inside host cells [49], [50]. Therefore, we examined whether *P. aeruginosa* causes cytoskeletal rearrangement during cell invasion through direct observation of bacteria and microfilaments in HMEECs by confocal laser microscopy. The cells were stained with rhodamine-phalloidin to visualize actin. Uninfected cells showed bright staining of actin cortical filaments and abundant stress fibers (Figure 6). However, within 15 minutes of infection with *P. aeruginosa*, microfilament redistribution was demonstrable as indicated by rounding of cells and small actin aggregates in conjunction with the adherent bacteria (Figure 6). With increase in post-infection time-period to 30 minutes there was further increase in actin condensation associated with bacterial binding sites (Figure 6). Bacteria were attached to HMEECs in groups, but individual adhered bacterium was also observable. Actin condensation was observable directly beneath the adherent bacteria. After 60 min post-infection, the actin cytoskeleton was observed to regain its original state despite the presence of intracellular bacteria. Interestingly, after internalization at 120 min post-infection, the bacteria were not surrounded by polymerized actin, suggesting that microfilament aggregation is required only during the initial stages of infection.

### OprF expression is required for invasion of HMEECs by *P. aeruginosa*

**Figure 7:**
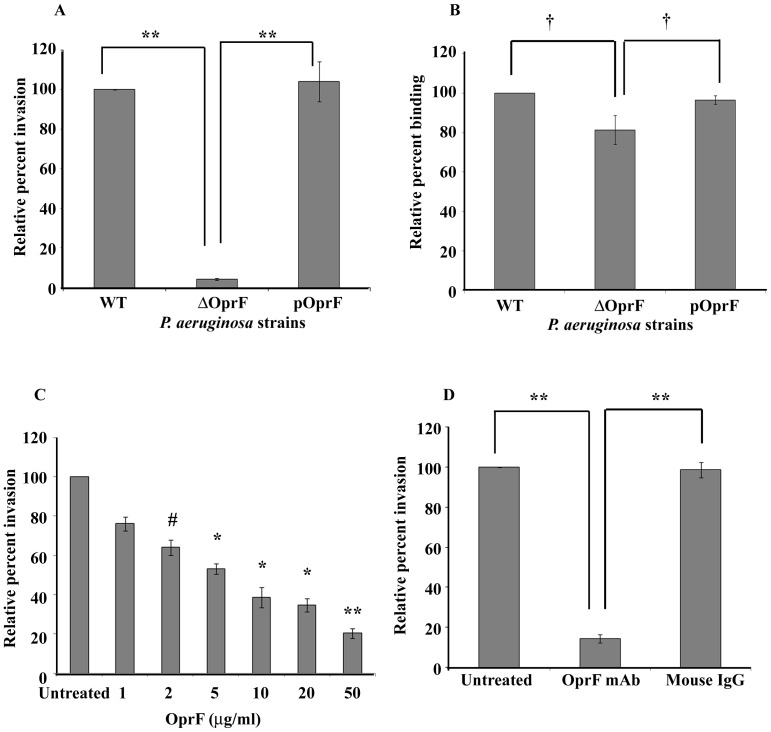
OprF expression in *P. aeruginosa* is required for HMEECs invasion. HMEECs were infected with *P. aeruginosa* strains at an MOI of 10 for 2h and bacterial invasion was determined by gentamicin protection assay (A). The binding of *P. aeruginosa* to HMEECs was also determined (B). In separate experiments HMEECs were pretreated with exogenous OprF (C) or bacteria were pretreated with anti-OprF monoclonal antibody (mAb) (D) and then performed invasion assay. Data represents mean ± SD. Results were expressed as percentage compared to the invasion or binding of the wild-type strain. **^#^** P<0.05 or * P<0.01 or ** P<0.001 or **^†^** P>0.05 compared to WT or pOprF.

Bacterial outer membrane proteins (OMPs) have been demonstrated to play an important role in interaction of pathogen with host cells [26]. Therefore, we investigated the role of OprF which is the most important OMP of *P. aeruginosa* in its ability to invade HMEECs. To test this, HMEECs were infected with wild-type (WT), Δ*OprF* mutant and *OprF* complemented (pOprF) strains of *P. aeruginosa* and cell invasion was assessed by gentamicin protection assay. There was 95% decrease in cell invasion of *OprF* mutant compared to WT strain (P<0.001) (Figure 7A). Interestingly, *OprF* complemented strain showed similar levels of cell invasion as the WT strain, suggesting that OprF plays an important role in invasion of HMEECs by *P. aeruginosa*. This inability of *OprF* mutant to invade HMEECs was not due to inefficient binding as no significant difference was observed between binding of WT, Δ*OprF* mutant and pOprF strains of *P. aeruginosa* (P>0.05) (Figure 7B).

To further confirm the role of OprF in cell invasion, we pre-treated HMEECs with different concentrations of exogenous purified OprF. There was a concentration dependent decrease in invasion of HMEECs by *P. aeruginosa*. Pretreatment of HMEECs with 1 μg/ml of exogenous OprF resulted in 17% decrease in bacterial invasion, whereas there was 75% decrease in invasion when cells were pretreated with 50 μg/ml of OprF compared to untreated cells (Figure 7C). Similarly, pretreatment of *P. aeruginosa* with anti-OprF monoclonal antibody (mAb) significantly decreased bacterial cell invasion compared to non-immune mouse IgG treated or untreated cells (P<0.001) (Figure 7D). These results suggest that OprF contributes to invasion of HMEECs by *P. aeruginosa*.

### Infection with *P. aeruginosa* causes cell damage

**Figure 8:**
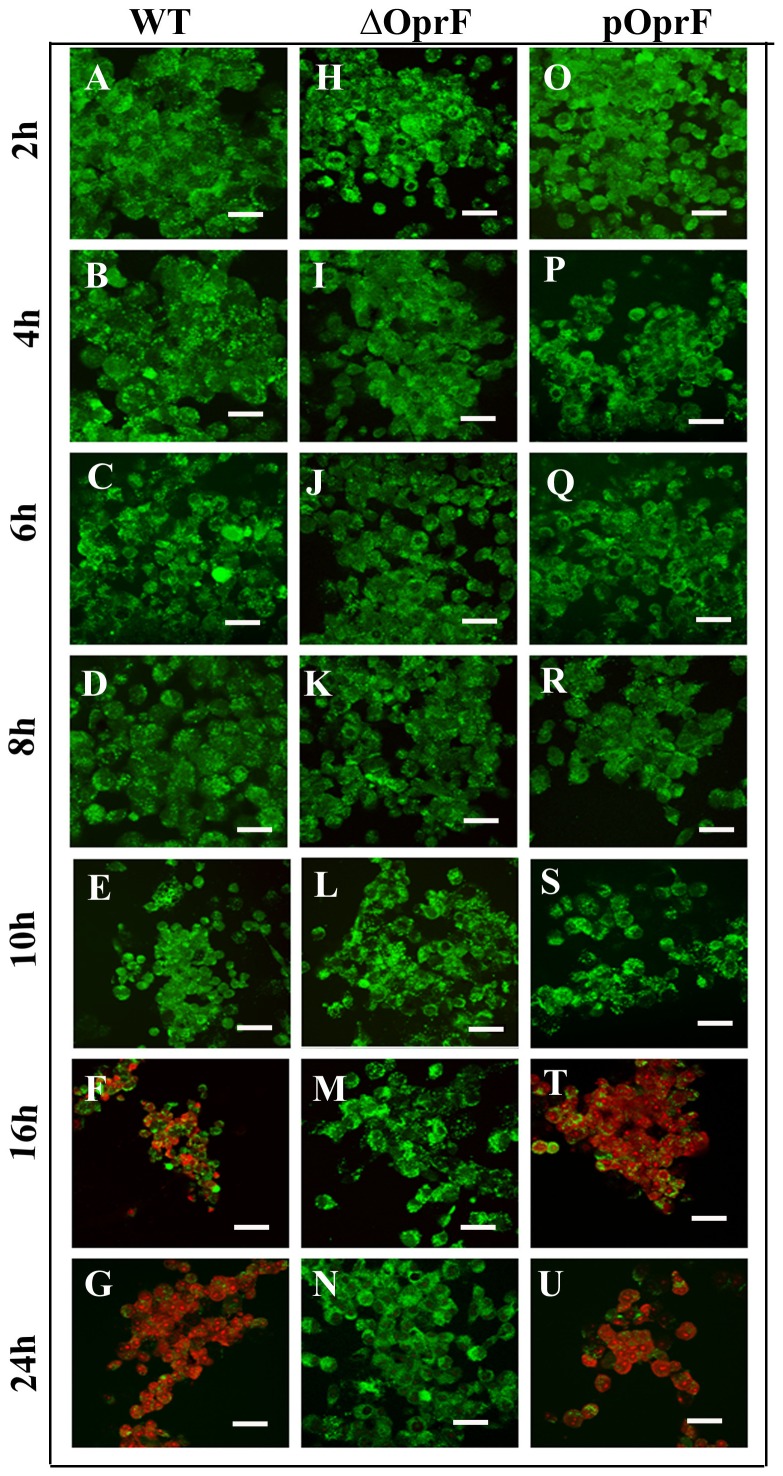
*P. aeruginosa* causes epithelial cell damage. HMEECs were infected with *P. aeruginosa* at an MOI of 10 for varying time periods. Cell damage was examined using the LIVE/DEAD fluorescent assay where uptake of green dye indicates live cells and red staining corresponds to dead cells. Results are representative of four independent experiments carried out in triplicate. Scale bars 10 μm.

Next, we determined whether *P. aeruginosa* infection of HMEECs causes cell damage. Cells were infected with bacteria for different time periods at different MOI and cell damage was assessed by a fluorescent dye based cell viability assay [51]. Examination of HMEECs by confocal microscopy showed significant differences in the interaction of dye with HMEECs. We observed that there was minimal cell damage up to 8h post-infection as indicated by the uptake of green dye and no red staining at an MOI of 1 and 10 in HMEECs infected with WT *P. aeruginosa* (Figure 8A-D and Figure S1A-D). However, HMECCs stained red and green after 8h post-infection, thereby suggesting significant cell damage after this time point (Figure 8E-G and Figure S1E-G). At higher MOI of 100, the cells stained green by 6h post-infection (Figure S2A-C). However, the cells stained red and green by 8h and 16h post-infection suggesting cell damage (Figure S2D-F). By 24h, complete destruction of the monolayers was observed with the predominant uptake of red dye by the cells (Figure S2G). Interestingly HMEECs took up the green dye with little or no evidence of cell death when infected with *OprF* mutant of *P. aeruginosa* at an MOI of 1 and 10 at all post-infection time periods (Figure 8H-N and Figure S1H-N). Even at higher MOI of 100 and 24h post-infection, the majority of the cells stained green demonstrating little or no cell death when infected with Δ*OprF P. aeruginosa* (Figure S2H-N). However, pOprF strain showed similar pattern of cell damage as WT strain at all MOIs and different time periods (Figure 8O-U and Figures S1O-U and S2O-U). HMEECs infected with pOprF strain of *P. aeruginosa* showed green staining by 8h post-infection followed by red and green staining at 10h, 16h and 24h post-infection.

**Figure 9:**
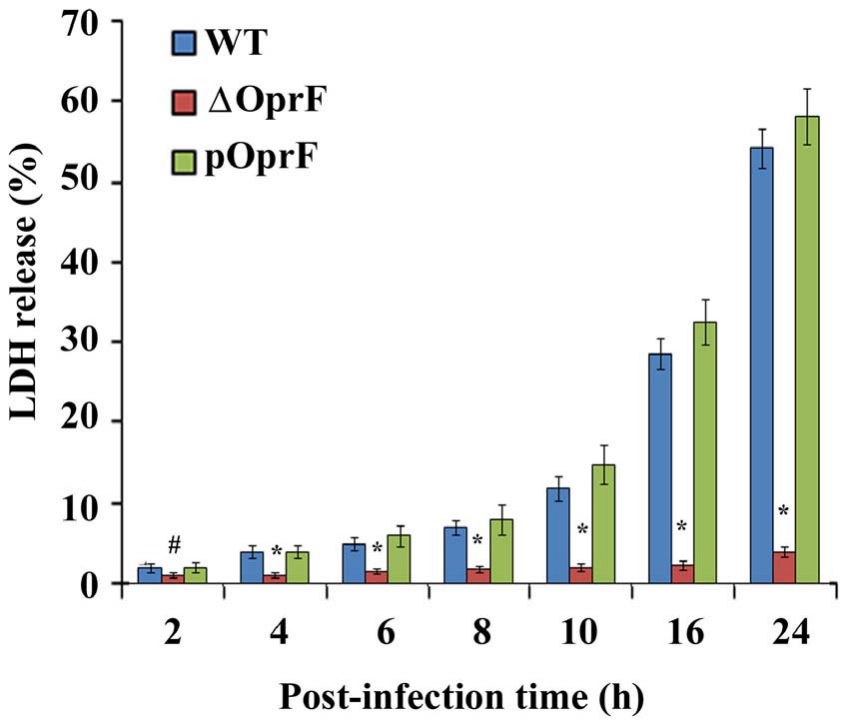
LDH release by HMEECs infected with *P. aeruginosa*. HMEECs were infected with *P. aeruginosa* at an MOI of 10 for varying time periods and LDH levels were determined in the culture supernatants of infected cells. Results were expressed as the percentage compared with maximum LDH release by lysed cells. Data represents mean ± SD and is representative of five individual experiments carried out in triplicate. # P<0.05 or *P<0.001 compared to WT or pOprF.

To further confirm these findings we determined LDH release in cell culture supernatants of HMEECs infected with *P. aeruginosa*. LDH release has been demonstrated to be a reliable biochemical indicator of cell damage in previous studies [46]–[48]. In agreement with confocal microscopy results, HMEECs infected with WT *P. aeruginosa* demonstrated minimal LDH release by 8h post-infection at an MOI of 1 and 10 (Figure 9 and Figure S3). However, by 10h post-infection, WT *P. aeruginosa* induced 8% LDH release by HMEECs at an MOI of 1, which reached to 20% and 39% by 16h and 24h post-infection, respectively (Figure S3). At an MOI of 10, LDH release was 12%, 29% and 55% at 10, 16 and 24h post-infection (Figure 9). At higher MOI of 100, LDH release was 30% and 58% at 10h and 16h respectively which increased to 75% by 24h post-infection (Figure S4). By contrast, Δ*OprF* mutant did not induce any significant LDH release above the baseline with any MOI tested and all post-infection time periods. However, pOprF strain induced similar levels of LDH release as WT bacteria. These results suggest that OprF expression in *P. aeruginosa* is required to cause cell damage.

## Discussion

*P. aeruginosa* is the most common pathogen causing CSOM [32]–[37]. CSOM is an important cause of hearing loss and is of serious concern, particularly in children, as it can cause developmental delays in speech and learning [4]. CSOM can also cause extra and intracranial complications [52]. The most frequent extracranial complications are subperiosteal abscess, facial paralysis, mastoiditis, and labyrinthitis [53]. The most common intracranial complications of CSOM are lateral sinus thrombosis, meningitis, cerebral abscess, otic hydrocephalus, extradural abscess, and encephalitis [54]. Since epithelium has been demonstrated to interact with pathogens [55], [56], it is likely that *P. aeruginosa* interacts with HMEECs in its ability to cause CSOM. However the specific nature of the interactions of *P. aeruginosa* with HMEECs has been unknown. In this study, we established an *in vitro* cell culture model using HMEECs to better understand the pathogenetic mechanisms utilized by *P. aeruginosa* during cell invasion.

The ability of pathogens to invade and persist in host cells is an important factor in their ability to cause disease [57]. The entry of pathogens into host cells provides a mechanism to evade immune defense mechanisms and cause infection [58]. We observed that *P. aeruginosa* is able to enter and survive inside HMEECs. SEM showed increase in bacterial number on the surface of HMEECs with increase in post-infection time. TEM demonstrated the sequence of events following interaction of *P. aeruginosa* with HMEECs including adherence of bacteria to the epithelial cell surface, formation of pseudopod-like structures around the adherent bacteria and subsequent internalization. In summary, the gentamicin protection assay and electron micrographs support the observations on the ability of *P. aeruginosa* to invade HMEECs. This may provide a safe niche to bacteria where it can persist, multiply, and potentially cause chronic infections like CSOM.

Pathogens harness cytoskeletal components to gain entry to, and to propel themselves within, host cells [49], [50]. In this study, we observed that *P. aeruginosa* induce a local bacterium-associated accumulation of polymerized actin during the invasion of HMEECs. This actin accumulation correlated with initial bacterial entry, after which point the cytoskeleton appears to assume its normal pattern of distribution. In addition, the entry of *P. aeruginosa* was inhibited by treatment of HMEECs with cytochalasin D, an inhibitor of actin polymerization and microfilament formation. Interestingly, there was a decrease in invasion following pretreatment with colchicine, nocodazole and vinblastine, each of which is an agent that blocks microtubule formation. This suggests that invasion of *P. aeruginosa* inside HMEECs involves both microfilament and microtubule dependent pathways. Utilization of host cell cytoskeleton is a common theme in microbial pathogenesis. Many invasive bacteria have common approaches of host cell interaction, but each species has evolved a subset of unique tactics that exploit normal host cell function, promoting survival and enhancing virulence. Some bacteria such as *Porphyromonas gingivalis*, *Edwardsiella* spp., *Neiserria gonorrhoeae*, enteropathogenic *Escherichia coli*, *Haemophilus influenzae*, and *Vibrio hollisae*, utilize both microfilaments and microtubules for entry [59]–[64]. The actin microfilaments have been shown to direct the engulfment of the bacteria by the host cell in *Salmonella* and *Shigella* infections [65], [66]. *Listeria* and vaccinia virus have also been shown to nucleate host cell actin on their surfaces to propel themselves through the host cell cytoplasm [67], [68]. However, this is the first study demonstrating *P. aeruginosa* induced cytoskeletal rearrangements during invasion of HMEECs.

Bacterial structures like outer membrane proteins (OMPs) play a crucial role in interaction of pathogens with host cells [26]. A significant finding of this study was that OprF is a critical microbial component responsible for the invasion of *P. aeruginosa* in epithelial cells. The cell invasion of *OprF* mutant was decreased by 95% in comparison to wild-type strain. Complementation with *OprF* restored the invasion property of *P. aeruginosa*, indicating the importance of OprF in cell invasion. However we observed no significant difference between binding of WT, Δ*OprF* and pOprF strains of *P. aeruginosa* to HMEECs suggesting OprF plays a little role in binding to these cells. In addition, pretreatment of HMEECs with exogenous OprF or pretreatment of *P. aeruginosa* with anti-OprF mAb significantly reduced bacterial cell invasion. These findings clearly implicate the role of OprF in invasion of HMEECs by *P. aeruginosa*. These results are in agreement with the observations reported with OprF homologue OmpA. OmpA has been shown to mediate invasion of meningitis causing *Escherichia coli* K1 and *Cronobacter sakazakii* inside human brain microvascular endothelial cells (HBMECs) [69], [70]. OmpA has also been implicated in astrocyte colonization by *E. coli* and cell invasion of *Acinetobacter baumanii* [71], [72]. All these studies, together with the results of the present investigation, highlight the crucial role of OMPs in the pathobiology of Gram-negative bacterial pathogens.

Pathogenic bacteria have the arduous task of interacting with host cells and reprogramming the complex molecular and cellular networks of these cells to allow bacterial replication and spread, while countering host-defense strategies. In this context, the interaction of pathogens with host cells has been demonstrated to induce cell damage [73]. Many pathogenic bacteria are equipped with a wide range of virulence determinants that interact with vital components of the host leading to cell damage [74], [75]. This causes release of intracellular bacteria and subsequent infection of neighboring cells. We observed that the interaction of OprF with HMEECs induces cell damage. Evidence for this role was provided by the findings that wild-type bacteria induced cell death whereas *OprF* mutant failed to cause cell damage. The OprF complemented strain showed similar levels of cell damage as the wild-type strain suggesting that OprF expression plays a crucial role in HMEECs damage.

In summary, our study shows that *P. aeruginosa* invades HMEECs that is dependent on both bacterial OprF expression as well as host microfilament and microtubule uptake mechanisms. The entry of *P. aeruginosa* subsequently causes cell damage *in vitro* that is similar to the *in vivo* damage observed in human patients. This *in vitro* cell culture model can be of immense importance in the characterization of the signal transduction pathways that lead to mucin overproduction and hence clinical manifestations of CSOM. This model can be used to identify host genes that are differentially expressed upon *P. aeruginosa* infection such as Toll–like receptors (TLRs). Microarray analysis of HMEECs infected with bacteria will provide novel information about key gene expression which might influence *P. aeruginosa* infection process. Our data provide novel insights in the pathogenesis of *P. aeruginosa* induced CSOM, and contribute to our understanding of invasion of HMEECs by bacteria. Understanding these host-pathogen interactions will enable the development of novel and effective therapeutic strategies to more efficiently treat CSOM and its sequelae.

## Supporting Information

Figure S1 ***P. aeruginosa*** **causes cell damage even at lower MOI.** HMEECs were infected with *P. aeruginosa* at an MOI of 1 for varying time periods and cell damage was assessed by fluorescent dye assay. The green color identifies viable cells whereas red color corresponds to dead cells. Results are representative of four independent experiments carried out in triplicate. Scale bars 10 μM.
(TIF)

Figure S2 **Epithelial damage infected with higher MOI of** ***P. aeruginosa*** **.** HMEECs infected with *P. aeruginosa* at an MOI of 100 for varying time-periods were subjected to LIVE/DEAD assay to examine cell damage. The viable cells uptake green dye whereas dead cells stain red. Results are representative of four independent experiments carried out in triplicate. Scale bars 10 μM.
(TIF)

Figure S3 **LDH release by HMEECs infected with** ***P. aeruginosa*** **at lower MOI.** LDH levels were determined in the culture supernatants of HMEECs infected with *P. aeruginosa* at an MOI of 1 for varying time periods. Data represents mean ± SD. Results are representative of four independent experiments carried out in triplicate. # P<0.05 or *P<0.001 compared to WT or pOprF.
(TIF)

Figure S4 **HMEECs infected with** ***P. aeruginosa*** **at higher MOI release substantial amounts of LDH.** LDH release by HMEECs infected with *P. aeruginosa* at an MOI of 100 was determined and expressed as percentage compared with maximum LDH release by lysed cells. Data represents mean ± SD and is representative of five individual experiments carried out in triplicate. # P<0.05 or *P<0.001 compared to WT or pOprF.
(TIF)

Video S1 **HMEECs were infected with** ***P. aeruginosa*** **at an MOI of 10 for 2h.** After incubation, samples were washed, fixed, and stained with anti-*P. aeruginosa* antibody followed by Alexa Fluor® 488 secondary antibody and subjected to confocal microscopy. Z-sections of confocal images were taken and generated a video using the ZEN software (Zeiss). The green color indicates bacteria and blue color is due to DAPI.
(AVI)
