# Supplementary figures and images for: In Vitro Interaction of Pseudomonas aeruginosa with Human Middle Ear Epithelial Cells
Source: PLoS One. 2014 Mar 14;9(3):e91885. doi: 10.1371/journal.pone.0091885 (PMC3954863; doi:10.1371/journal.pone.0091885)

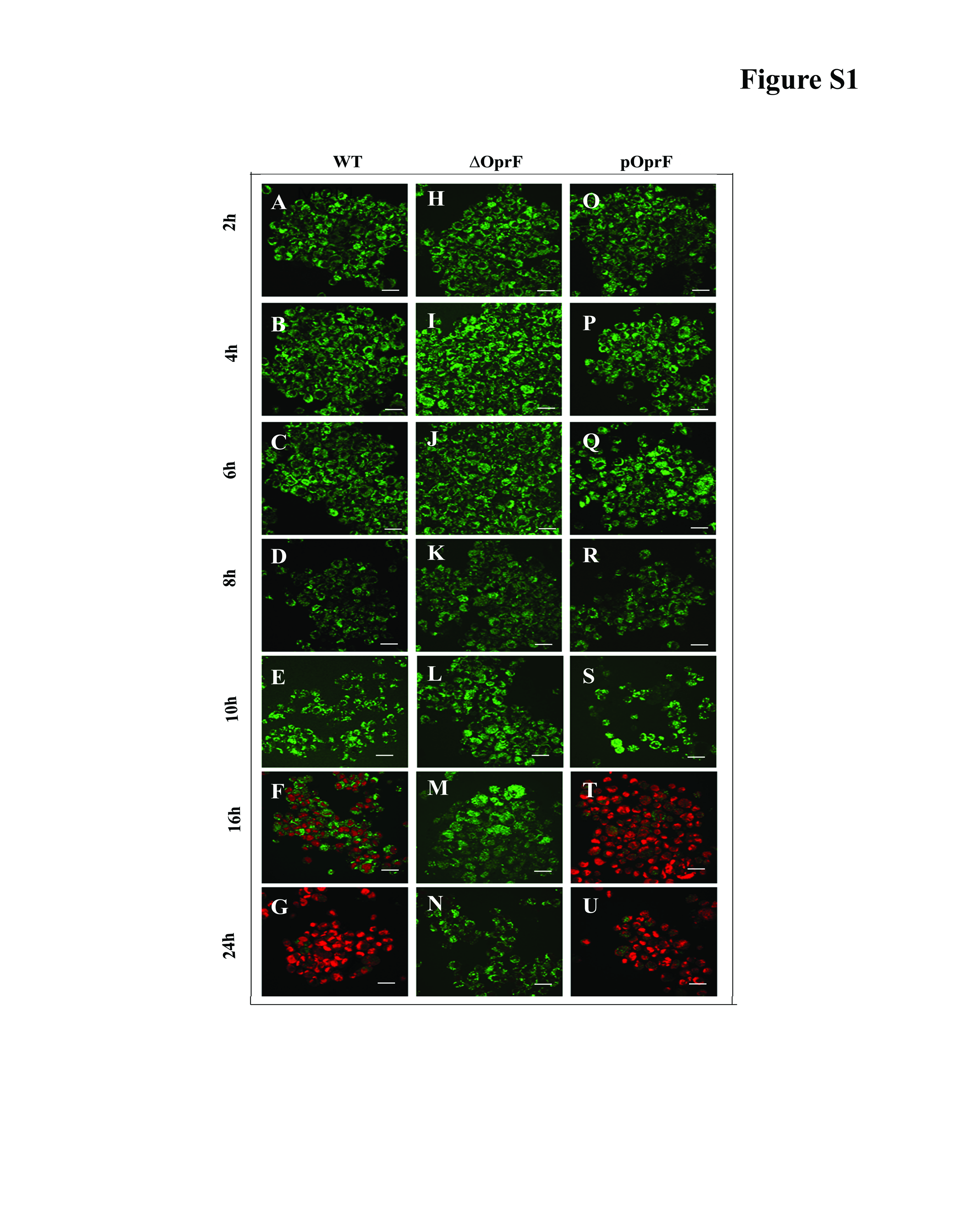

Supplement: Figure S1 — P. aeruginosa causes cell damage even at lower MOI. HMEECs were infected with P. aeruginosa at an MOI of 1 for varying time periods and cell damage was assessed by fluorescent dye assay. The green color identifies viable cells whereas red color corresponds to dead cells. Results are representative of four independent experiments carried out in triplicate. Scale bars 10 μM. (TIF) [file pone.0091885.s001.tif]

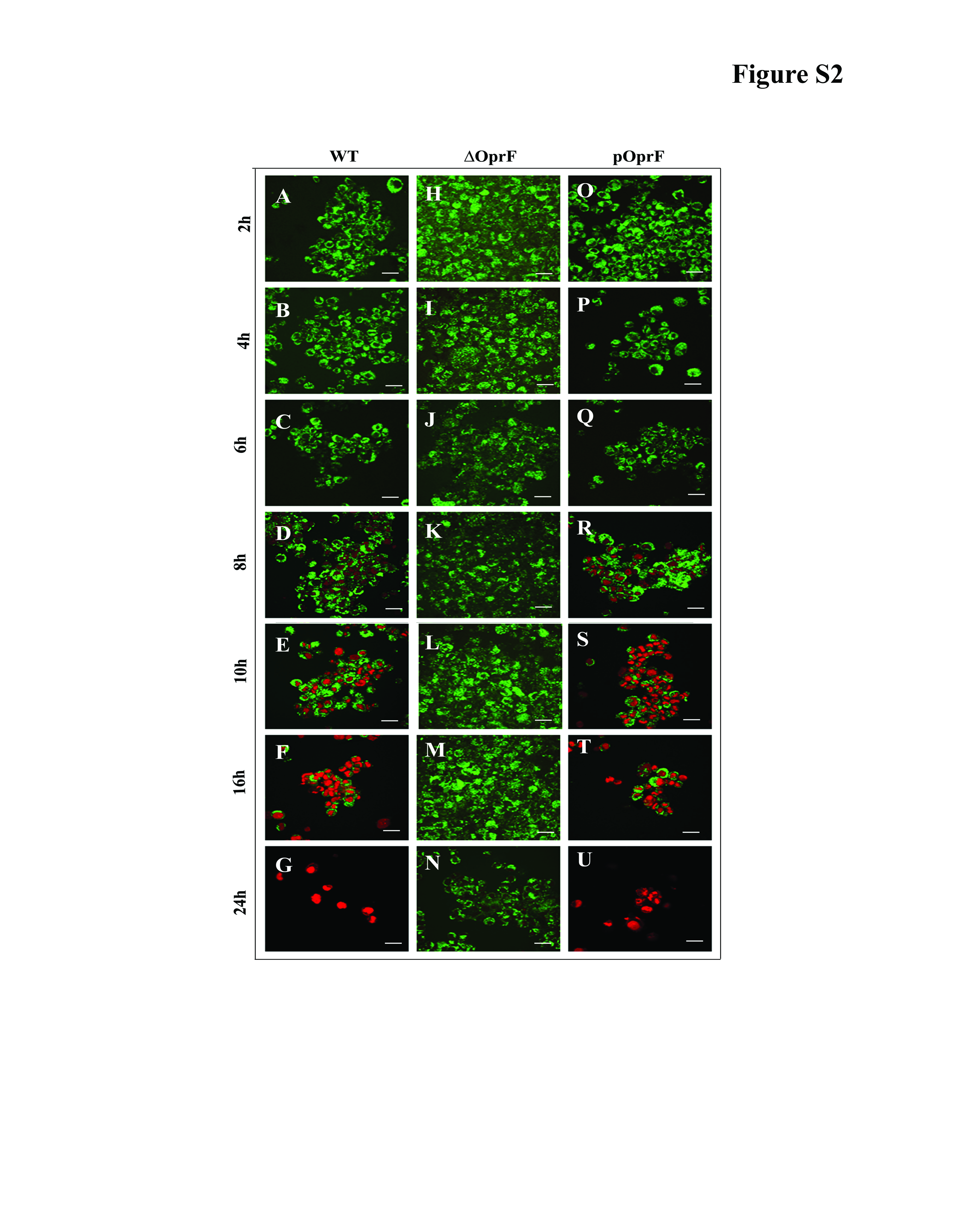

Supplement: Figure S2 — Epithelial damage infected with higher MOI of P. aeruginosa . HMEECs infected with P. aeruginosa at an MOI of 100 for varying time-periods were subjected to LIVE/DEAD assay to examine cell damage. The viable cells uptake green dye whereas dead cells stain red. Results are representative of four independent experiments carried out in triplicate. Scale bars 10 μM. (TIF) [file pone.0091885.s002.tif]

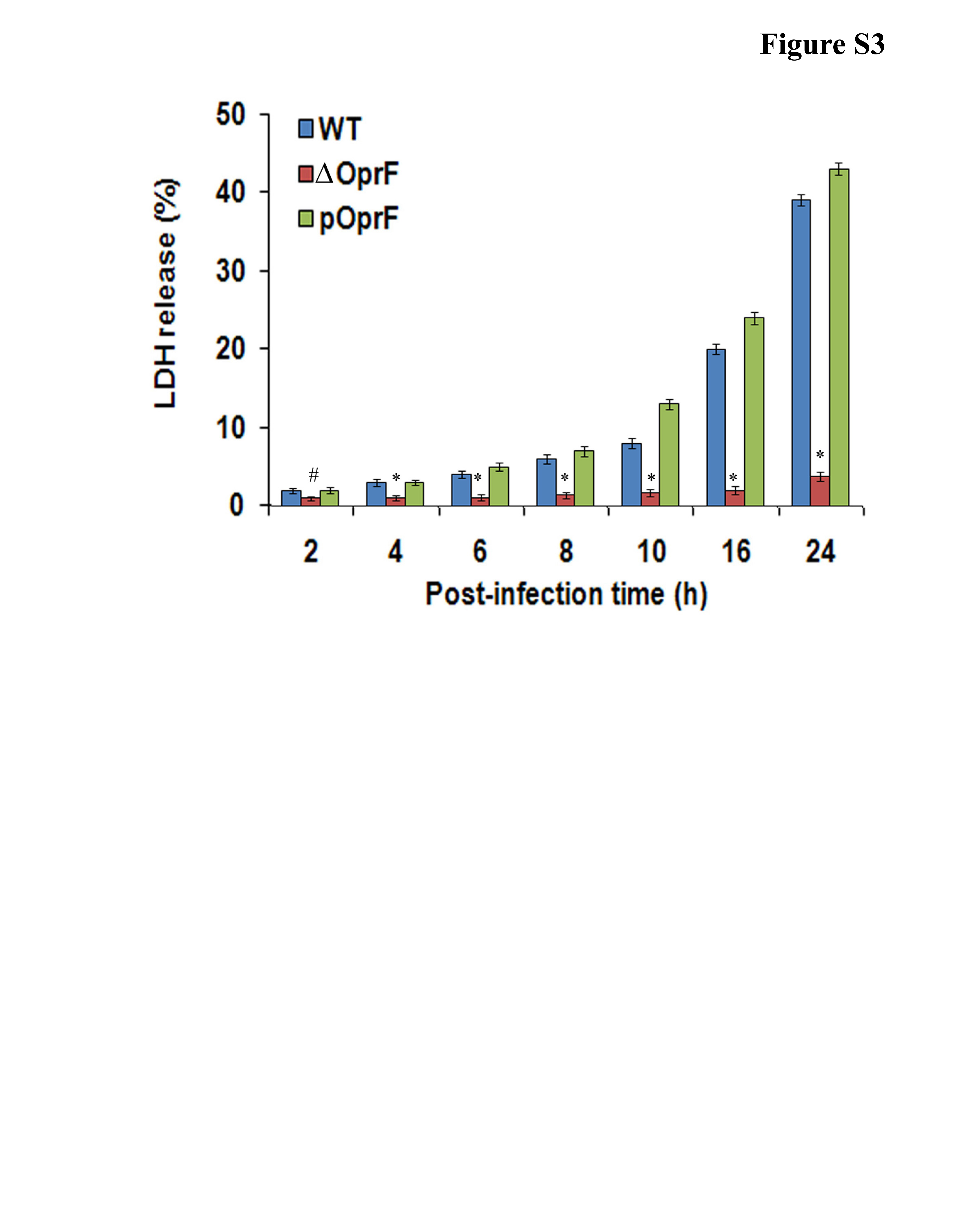

Supplement: Figure S3 — LDH release by HMEECs infected with P. aeruginosa at lower MOI. LDH levels were determined in the culture supernatants of HMEECs infected with P. aeruginosa at an MOI of 1 for varying time periods. Data represents mean ± SD. Results are representative of four independent experiments carried out in triplicate. # P<0.05 or *P<0.001 compared to WT or pOprF. (TIF) [file pone.0091885.s003.tif]

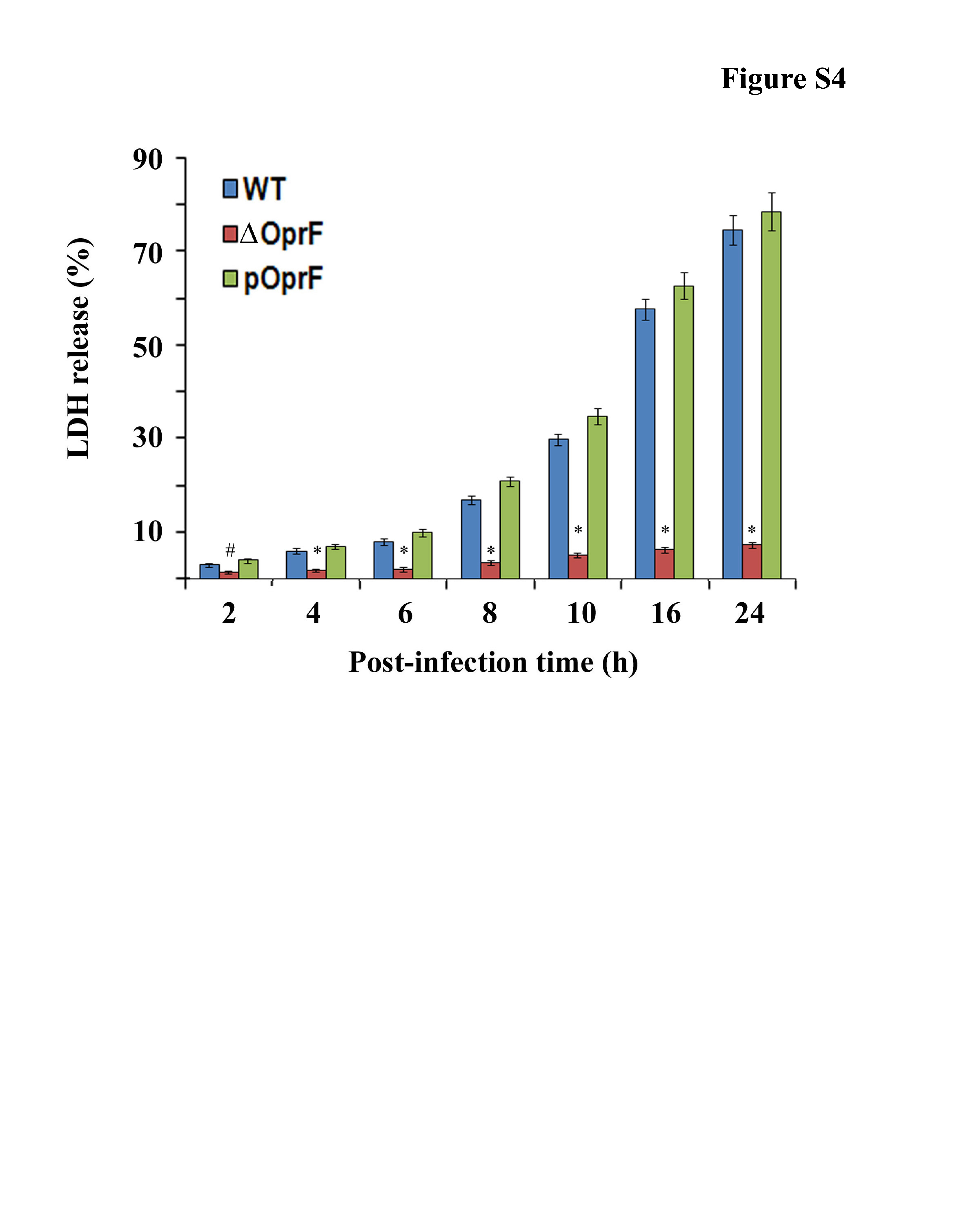

Supplement: Figure S4 — HMEECs infected with P. aeruginosa at higher MOI release substantial amounts of LDH. LDH release by HMEECs infected with P. aeruginosa at an MOI of 100 was determined and expressed as percentage compared with maximum LDH release by lysed cells. Data represents mean ± SD and is representative of five individual experiments carried out in triplicate. # P<0.05 or *P<0.001 compared to WT or pOprF. (TIF) [file pone.0091885.s004.tif]
